# Use of Autoantibodies to Detect the Onset of Breast Cancer

**DOI:** 10.1155/2014/574981

**Published:** 2014-07-21

**Authors:** Jérôme Lacombe, Alain Mangé, Jérôme Solassol

**Affiliations:** ^1^INSERM-U896, Montpellier Cancer Research Institute (IRCM), 34298 Montpellier Cedex 5, France; ^2^Department of Biopathology, CHU Montpellier, 34295 Montpellier Cedex 5, France; ^3^University of Montpellier I, 34000 Montpellier, France; ^4^Montpellier Cancer Institute (ICM), Department of Clinical Oncoproteomics, 34298 Montpellier Cedex 5, France

## Abstract

The widespread use of screening mammography has resulted in increased detection of early-stage breast disease, particularly for *in situ* carcinoma and early-stage breast cancer. However, the majority of women with abnormalities noted on screening mammograms are not diagnosed with cancer because of several factors, including radiologist assessment, patient age, breast density, malpractice concerns, and quality control procedures. Although magnetic resonance imaging is a highly sensitive detection tool that has become standard for women at very high risk of developing breast cancer, it lacks sufficient specificity and costeffectiveness for use as a general screening tool. Therefore, there is an important need to improve screening and diagnosis of early-invasive and noninvasive tumors, that is, *in situ* carcinoma. The great potential for molecular tools to improve breast cancer outcomes based on early diagnosis has driven the search for diagnostic biomarkers. Identification of tumor-specific markers capable of eliciting an immune response in the early stages of tumor development seems to provide an effective approach for early diagnosis. The aim of this review is to describe several autoantibodies identified during breast cancer diagnosis. We will focus on these molecules highlighted in the past two years and discuss the potential future use of autoantibodies as biomarkers of early-stage breast cancer.

## 1. Introduction

Breast cancer is the most common malignancy and the second most common cause of cancer-related mortality in women [1]. Successful strategies for screening, early diagnosis, prognosis, and risk stratification are needed to decrease mortality and increase the probability of curing the disease. Currently, mammography is the gold standard of breast cancer screening and remains the only screening test proven to reduce mortality. However, not all cancers can be visualized on screening mammograms. Indeed, mammographic sensitivity decreases significantly as breast density increases, with sensitivity reported to be as low as 45% in women with extremely dense breasts [2]. Conversely, mammography can also lead to overdiagnosis (i.e., detection of tumors that might not need intervention) and can lead to unnecessary treatment of some patients [3]. Therefore, considerable efforts have been undertaken to produce an effective screening method for early-stage breast cancer. Both full-field digital mammography and computer-aided detection programs have been proposed, but results from these methods remain controversial [4]. The ability of magnetic resonance imaging (MRI) to detect the presence and extent of small tumors seems to exceed that of both mammography and ultrasound, with low specificity. However, additional investigations are still required to confirm this finding [5]. Finally, improving early-stage breast cancer screening is needed, particularly for women with high breast density [6].

## 2. Current Biomarkers and Clinical Utility of Autoantibodies 

For early detection to be an effective and practical approach, screening tests must satisfy four basic requirements. First, screening tests should be able to distinguish healthy individuals from cancer cases with a high degree of accuracy, sensitivity, and specificity (namely, low false-negative and false-positive rates). Second, detection should be possible before the disease progresses to an advanced stage, or even prior to the first manifestation of clinical signs, when it is still curable. Third, the test should ideally allow discrimination between lesions that are aggressive and require treatment and those that ultimately will do no harm, thus reducing the problem of overdiagnosis. Fourth, tests should be inexpensive and well accepted by the target population [7]. Breast cancer markers currently in use do not satisfy all these requirements. Therefore, FDA-approved protein tumor markers currently used in clinical practice, such as circulating tumor cell (CTC) proteins, estrogen receptor (ER), progesterone receptor (PR), Her-2/neu, CA 15-3, and CA27.29, are not approved for screening or early diagnosis [8]. Rather, ER and PR assays are recommended for prognosis and determining response to therapy, while Her-2/neu is used to assess appropriate therapeutic options. Moreover, ER, PR, and Her-2/neu measurements are based on immunohistochemistry, a method that requires invasive intervention such as biopsy to obtain samples. The level of CTCs is reported to be associated with cancer progression and survival. Finally, CA15-3 and CA27.29 serum biomarkers are only recommended for monitoring disease state and response to therapy.

Development of a sensitive, specific, and reproducible assay to identify biomarkers that can accurately determine the onset of breast cancer, particularly noninvasive tumors, is an attractive goal. This assay should be applicable for routine clinical use, require minimal time, and present little risk for the patient (e.g., venipuncture). Based on these criteria, autoantibodies have great potential as breast cancer biomarkers. Indeed, autoantibodies are secreted and are therefore easily accessible. Autoantibodies are present in sera before tumor-associated antigens (TAAs) can be detected. Autoantibodies also correspond to efficient biological amplification of TAAs and are secreted into serum prior to first clinical signs. Moreover, antibodies are highly stable in serum samples and, unlike other polypeptides, are not subject to proteolysis, simplifying sample handling. They have a long lifetime in blood (*T*_1/2_ between 7 to 30 days, depending on the subclass of immunoglobulin) and may persist as long as a corresponding autoantigen that elicited the original specific humoral response. Finally, antibodies are biochemically well-characterized and many reagents and techniques are available for their detection, simplifying assay development [9].

## 3. First Steps toward the Identification of Autoantibodies in Cancer Patients

In 1955, Baldwin was the first to demonstrate that the immune system could react to a developing tumor [10]. He showed that tumor extracts could cause considerable tissue destruction when injected into growing tumors in rats. Tumors in these rats regressed, and the animals remained free of recurrent tumor growth. Furthermore, injected rats were found to be immune to subsequent implants of the same tumor. These results suggested that the development of tumor immunity depends upon the presence of immunogenetic differences in the tumor-host relationship. Also in 1955, Graham J. B. and R. M. Graham screened autoantibodies in the sera of 48 patients with gynecological cancers including cervical, ovarian, and uterine lesions using the complement-fixation technique [11]. Twelve of these samples demonstrated significant autoantibody titers, suggesting for the first time that autoantibodies could be used as a diagnostic tool for cancer. In 1970, Taylor and Odili identified the first neoantigen, which was highly similar to the T antigen of oncogenic DNA virus, eliciting a specific humoral response in breast cancer [12]. In the following years, using immunofluorescence approaches Priori et al. confirmed the presence of autoantibodies in random sera from breast cancer patients [13]. In 1975, Wasserman et al. demonstrated that the incidence of autoantibodies at diagnosis of breast carcinoma was higher in patients who developed local recurrences or distal metastases within 2 years than in patients free from recurrence [14]. Although these results have not been be confirmed, this study was the first to use autoantibodies as prognostic biomarkers.

## 4. Autoantibodies to Individual TAAs

**Figure 1:**
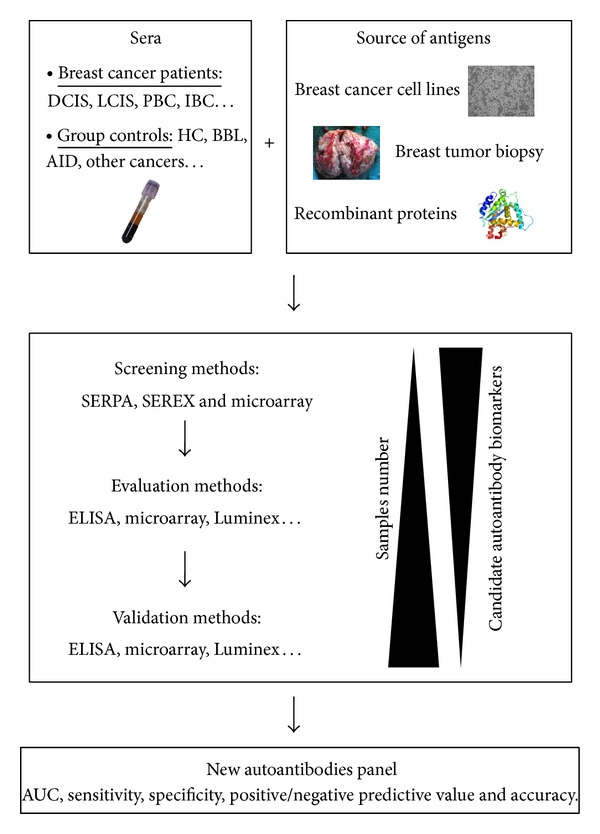
Breast cancer autoantibody research pipeline. Methodologies for identification of TAAs consist to locate specific immunogenic proteins specific from a source of antigens (recombinant protein or extracted from cell cultures or tumors). Different screening methods have been developed in order to identify TAAs such as SEREX (serological identification of antigens by recombinant expression cloning), SERPA (serological proteome analysis), or more recently microarray. TAAs were subsequently evaluated and validated using ELISA on multiplex methodologies such as Luminex or microarray. DCIS (ductal carcinoma* in situ*); LCIS (lobular carcinoma* in situ*); PBC (primary breast cancer); IBC (invasive breast cancer); HC (healthy control); BBL (benign breast lesions); AID (autoimmune disease); AUC (area under curve).

Considerable efforts have been made to identify autoantibodies and their antigen counterparts to detect and/or monitor cancer progression. Over the past 10 years, several technical approaches have been developed (Figure 1), and many studies have demonstrated the potential use of autoantibodies for early breast cancer detection. These molecules included p53, MUC-1, heat shock proteins (HSP-27, HSP-60, and HSP-90), HER2/neu/c-erg B2, GIPC-1, c-myc, c-myb, cancer-testis antigens (NY-ESO-1), BRCA1, BRCA2, endostatin, lipophilin B, cyclin B1, cyclin D1, fibulin, insulin-like growth factor binding protein 2 (IGFBP-2), topoisomerase II alpha (TOPO2*α*), and cathepsin D (for review see [9, 15]). Recently, an original study aimed to address the temporal relationship between breast cancer development and serum antibody responses against two previously identified TAAs (p53 and HER-2/neu) with sera collected prior to diagnosis, at diagnosis, and during treatment [16]. At the time of treatment, p53 and HER-2/neu autoantibodies were significantly increased in the sera collected from patients with breast cancer. Interestingly, comparison of antibody responses in prediagnostic samples and controls demonstrated that HER2/neu and p53 antibodies can be detected in sera collected, on average, more than 150 days prior to diagnosis. Although sample sizes were relatively small (33 cases and 45 controls), and although the percentage of patients producing autoantibodies against HER2 and p53 in prediagnostic samples was also low (15% and 6%, resp.), these results confirm the potential usefulness of these markers as indicators of the early stages of carcinogenesis.

In the past two years, new autoantibodies have been identified. Sun et al. provided the first evidence for the presence of circulating SOX2 antibodies in breast cancer [17]. The authors determined the expression levels of SOX2 antibodies in sera from 282 breast cancer patients, 78 benign breast disease patients, and 194 healthy women, using indirect enzyme-linked immunosorbent assay (ELISA). The results showed that SOX2 antibodies were more prevalent in patients with breast cancer (18.4%) than in healthy women (2.6%, *P* < 0.0001) and patients with benign breast disease (6.4%, *P* = 0.011). Based on the concentration of circulating SOX2 antibodies, the investigators were able to discriminate between breast cancer patients and healthy controls (*P* < 0.001) and between breast cancer patients and those with benign breast disease (*P* < 0.001). Furthermore, in breast cancer patients the prevalence of SOX2 antibodies was associated with higher tumor grade and positive nodal status. Liu et al. also identified a specific humoral response against the p90/CIP2A antigen [18]. In 256 sera samples (168 from breast cancer patients and 88 from normal individuals), the authors showed that p90/CIP2A elicited higher autoantibody production in breast cancer (19.1%) than in normal volunteers (2.3%). These results were supported by the higher frequency of p90/CIP2A expression in breast cancer tissues than in adjacent normal tissues. Ye et al. have assessed the levels of CD25 and FOXP3 autoantibodies levels, previously identified in lung [19] and esophageal cancer [20, 21], in 152 breast cancer patients and 112 healthy individuals [22]. No significant differences were observed between breast cancer patients and controls. However, patients with stage I primary breast cancer exhibited higher expression of CD25 autoantibodies than healthy controls. In addition, Heo et al. observed by ELISA that a mimotope for circulating anti-cytokeratin 8/18 autoantibody discriminated breast cancer patients from normal subjects with a sensitivity and a specificity of 50% and 82.61%, respectively [23].

## 5. Tailor-Made Autoantibody Panels in Breast Cancer

Usually, only 10–30% of cancer patients elicited a specific humoral response against a single TAA [24]. The reason for this low sensitivity could lie in the heterogeneous nature of breast cancer, whereby different proteins are aberrantly processed or regulated in patients with the same type of cancer [25]. Therefore, several studies have evaluated the usefulness of detecting various autoantibodies as a panel to increase the accuracy of a potential diagnostic test (Table 1). Chapman et al. were the first to assess the frequency of seven autoantibodies (p53, c-Myc, HER2, NY-ESO-1, BRCA1, BRCA2, and MUC1) in a ductal carcinoma* in situ* (DCIS) population of 40 patients [26]. Interestingly, reproducibly elevated serum levels of autoantibodies were seen in at least one of the six antigens in 64% of primary breast cancer patients and 45% of patients with DCIS, at a specificity of 85%. Desmetz et al. reported a multimarker signature combining HSP60, MUC1, FKBP52, PPIA, and PRDX2 that reached sensitivity, specificity, and accuracy of 72.2, 72.6, and 72.4%, respectively, in DCIS compared with healthy individuals [27]. Recently, the same group identified a panel of five new autoantibodies from 80 subjects (20 patients with early-stage DCIS or primary breast cancer, 20 women with benign breast lesions, 20 healthy controls, and 20 women with autoimmune disease) [28]. This panel consisted of GAL3, PAK2, PHB2, RACK1, and RUVBL1. The expression levels of these five markers were validated by ELISA on a second set of sera (182 patients: 59 patients with primary breast cancer, 55 patients with DCIS, and 68 healthy controls). The signature significantly discriminated early-stage cancer from healthy individuals (AUC = 0.81; 95% CI: 0.74–0.86). Interestingly, this value was high in both node-negative early-stage primary breast cancer patients (AUC = 0.81; 95% CI: 0.72–0.88) as well as in DCIS patients (AUC = 0.85; 95% CI: 0.76–0.95). Using microarray, Ye et al. assessed Imp1, p62, Koc, p53, cmyc, survivin, p16, cyclin B1, cyclin D1, and CDK2 autoantibody levels in 122 patients [29]. The antibody frequency to the individual TAAs in breast cancer was variable and ranged between 7.3% and 22.0%. However, with the successive addition of TAAs to a total of eight antigens, there was a stepwise increase in positive antibody reactions, reaching a sensitivity of 61.0% and a specificity of 89.0% in breast cancer. The positive and negative likelihood ratios were 5.545 and 0.438, respectively, which showed that the clinical diagnostic value of a parallel assay of eight TAAs was high. Moreover, the positive predictive value (PPV) was 73.5% and the negative predictive value (NPV) was 82.0%. Using a T7 breast cancer complementary deoxyribonucleic acid phage library for tumor-associated antigens, Dong et al. identified hnRNPF and FTH1 autoantibodies in breast cancer [30]. Autoantibodies have been evaluated by ELISA in 150 breast cancer, 150 normal, and 40 non-breast-cancer serum samples. Autoantibodies were significantly higher in breast cancer patients relative to controls (*P* < 0.01), with an AUC of 0.73 and 0.69 for hnRNPF and FTH1 autoantibodies, respectively. Specificities remained relatively low (56.1% for FTH1 and 60.8% for hnRNPF autoantibodies). Even when the two biomarkers were combined, the specificity remained low (72.0%), while the sensitivity increased to 91.0%. However, when both of these autoantibody biomarkers combined with serum CA 15-3 values, the AUC increased to 0.93, with 89.3% sensitivity and 93.8% specificity.

Autoantibody assessment as a prognostic biomarker has been poorly investigated. Using protein microarrays, Mangé et al. described significant and consistent differences in the level of autoantibodies targeting specific antigens in a population of 20 patients with DCIS and 20 with early-stage breast cancer [35]. In this protein microarray experimental study, a set of five autoantibody targets (RBP-Jk, HMGN1, PSRC1, CIRBP, and ECHDC1) with the highest differential signal intensities was used to establish an autoantibody signature of the transition from DCIS to early-stage cancer. The performance of this humoral signature was then assessed in an independent set of 120 newly diagnosed patients using ELISA. The results showed that this signature could significantly discriminate DCIS from invasive breast cancer (AUC = 0.794; 95% CI: 0.674–0.877). Moreover, this panel could clearly distinguish low-grade DCIS from high-grade DCIS (AUC = 0.749; 95% CI: 0.581–0.866). Interestingly, the autoantibody signature could significantly divide the DCIS patients into groups with either poor prognosis or good prognosis (*P* = 0.01). Taken together, these results suggested that examining the humoral response to preinvasive lesions could identify potential markers that accurately detect DCIS patients at high risk for subsequent local recurrence.

## 6. Clinical Implications

Until now, a wide range of autoantibodies has been identified. Although several studies present hopeful preliminary results, there is a need to validate autoantibody signatures on a large prospective population. Indeed, for biomarkers reach to the clinic, their original performance must be independently reproduced in subsequent validation studies [36]. However, most of the studies cited above are limited by the size of the validation sample set. As an example in a breast cancer population, when MUC1 autoantibody was assessed in prediagnostic sera from over 2000 women, distributed across one discovery set (273 cases versus 273 controls) and two validation sets (426 cases versus 426 controls and 303 cases versus 606 controls), no differences could be observed between cases and controls. This result demonstrates the need to validate results in several independent cohorts. Validation is usually performed by ELISA, an assay that is rapid and simple to carry out and can handle a large number of samples in parallel. However, multiplex analysis remains difficult because ELISA processing is usually time consuming and expensive. Currently, two types of techniques allow for multiplex analysis. The Lumina immunobead platform (LabMAP, FlowMetrix) uses digital signal processing capable of classifying polystyrene beads (microspheres) dyed with distinct proportions of red and near-infrared fluorophores. These proportions define a “spectral addresses” for each bead population. As a result, up to one hundred different detection reactions can be carried out simultaneously on the various bead populations in very small sample volumes [37]. This technology has already been utilized in non-small-cell lung cancer autoantibodies detection [38, 39]. In 2009, Kim et al. used the bead array platform for discovering signatures specific to primary nonmetastatic breast cancer and differentiating these patients from normal subjects using sensitive combinatorial classifiers [40]. In his work, an antibody bead array of 35 markers was constructed, and an initial study population consisting of 98 breast cancer patients and 96 normal subjects was analyzed. Multivariate classification algorithms were then used to find discriminating biomarkers, which were validated with another independent population of 90 breast cancer subjects and 79 healthy controls. Serum concentrations of three autoantibodies (against epidermal growth factor, soluble CD40-ligand, and proapolipoprotein A1) were increased in breast cancer patients, whereas five autoantibodies (against high-molecular-weight-kininogen, apolipoprotein A1, soluble vascular cell adhesion molecule-1, plasminogen activator inhibitor-1, vitamin-D binding protein, and vitronectin) were decreased. The classifier was able to discriminate breast cancer patients from the normal population with high accuracy (87.6% to 91.8% according to classification method). The second multiplex approach consists of protein microarray technology. This technique was developed for high throughput and multiparametric assays that allow for the identification of multiple tumor markers. Combining various TAAs onto microstructured microarray under optimized conditions (spotting pH buffer, surface chemistry, and blocking procedure) could improve sensitivity and specificity of anti-TAA autoantibody detection. A recent paper showed the utility of protein microarray for autoantibody detection in sera of breast cancer patients [41]. The authors investigated both surface chemistry and protein immobilization conditions to improve sensitivity of the detection of tumor autoantibodies on these microarrays. Ten proteins (CEA, p53, HER2, NY-ESO-1, Hsp60, Hsp70, MYCL1, CHEK2, HNRNPK, and NME1) were immobilized onto microstructured glass slides functionalized with six different surface chemistries to detect autoantibodies in sera of breast cancer patients. The authors demonstrated that there is not a unique surface chemistry suitable for all proteins and that immobilization parameters must be optimized for each protein. Thus, to validate the best surfaces for protein immobilization and biological activity, sera from 29 breast cancer patients and 28 healthy donors were tested on TAA microarrays. Through a combination of five TAAs (Hsp60, p53, Her2-Fc, NY-ESO-1, and Hsp70) immobilized on an optimized surface chemistry, 82.7% of breast cancer patients were specifically detected. The potential cost and time savings that could be realized by using these technologies relative to other methods provide a strong impetus for their routine use in both research and clinical settings. Nevertheless, as with all clinical laboratory tests, questions of reproducibility, precision, and accuracy must be addressed to validate these assays [37].

With the inherent heterogeneity of breast tumors and our limited understanding of the humoral immune response to cancer, there are some obstacles to autoantibody identification and their routine clinical use for early breast cancer detection. However, this promising type of diagnostic strategy should continue to be developed. Recent published reports indicate an encouraging future for the implementation of sensitive and specific tests. It is conceivable that a humoral signature based on the detection of specific autoantibodies can be applied to the detection of cancer as well as to the tracking of disease progression and response to therapy. The most significant hope may be the use of such a signature for detection of cancers to which patients are predisposed by monitoring autoantibody profiles before the first clinical manifestation of symptoms. Finally, investigators should pursue a transition from the current system of retrospective studies to prospective analyses of patients' autoantibody responses and an assessment of this method's efficacy in clinical settings.

## Figures and Tables

**Table 1:** Comparison between individual autoantibodies and autoantibody signatures in breast cancer.

TAA composition	Serum samples	Methods	Individual autoantibodies			Panel			Ref.
			AUC	Sensitivity	Specificity	AUC	Sensitivity	Specificity	
p53, c-myc, HER2, NY-ESO-1, BRCA1, BRCA2, and MUC1	40 DCIS versus 97 PBC versus 90 HC	ELISA	—	24; 13; 18; 26; 8; 34; 20% (PBC versus HC) 15; 8; 13; 8; 3; 23; 13% (DCIS versus HC)	96; 97; 94; 94; 91; 92; 98%	—	64% (PBC versus HC) 45% (DCIS versus HC)	85%	[26]

p53, p62, c-myc, cyclin B1, survivin, and IMP1	64 breast cancers versus 346 HC	ELISA	Frequency of AABs to seven cancer-associated antigens: 7.8; 7.8; 18.8; 4.7; 7.8; 7.8% (breast cancer) and 1.4; 2; 1.2; 1.7; 2; 2% (HC)			Frequency of AABs to any seven antigens: 43.8% (breast cancer) and 10.1% (HC)			[31, 32]
						—	67–92%	85–95%

MUC1, cyclin D1, cathepsin D, p53, HER2, IGFBP-2, and TOPO2*α*	184 late-stage IBC versus 134 HC	ELISA	Frequency of AABs to seven cancer-associated antigens: 20; 8; 5; 10; 13; 14; 7% (breast cancer) and 3; 5; 3; 1; 5; 1; 3% (HC)			Frequency of AABs to any p53. HER2. MUC1 and TOPO2*α* antigens: 31% (breast cancer)			[33]
			0.48 (p53)	—	—	0.61 (p53 + HER2) 0.63 (p53 + HER2 + IGFBP-2 + TOPO2*α*)	—	—	

ASB-9, SERAC1, and RELT	*Training set*: 5 breast cancers versus 5 HC	SEREX	0.593; 0.642; 0.727	41; 47; 53% (training set) 59; 53; 65% (validation set)	100; 100; 100% (training set) 65; 71; 77% (validation set)	0.861	80% (training set) 77% (validation set)	100% (training set) 82.8% (validation set)	[34]
	*Validation set*: 87 breast cancers versus 87 HC	ELISA							

HSP60, MUC1, FKBP52, PPIA, PRDX2, HSP60, and MUC1	*Training set*: 20 breast cancers versus 20 other cancers versus 20 AID versus 20 HC	SERPA	0.69; 0.69; 0.66; 0.57; 0.59 (HC versus cancer) 0.63; 0.68; 0.66; 0.64; 0.63 (HC versus early-stage PBC) 0.73; 0.70; 0.65; 0.51; 0.56 (HC versus DCIS)	35; 37; 50; 50; 45% (HC versus cancer) 44; 43; 43; 48; 45% (HC versus early-stage PBC) 27; 32; 55; 51; 45% (HC versus DCIS)	87; 88; 87; 87; 86% (HC versus cancer) 86; 87; 90; 89; 87% (HC versus early-stage PBC) 90; 89; 87; 86; 84% (HC versus DCIS)	0.74 (HC versus cancer) 0.73 (HC versus early-stage PBC) 0.80 (HC versus DCIS)	60.5% (HC versus cancer) 55.2% (HC versus early-stage PBC) 72.2% (HC versus DCIS)	77.2% (HC versus cancer) 87.9% (HC versus early-stage PBC) 72.6% (HC versus DCIS)	[27]
	*Validation set*: 82 DCIS versus 60 early-stage PBC versus 93 HC	ELISA							

GAL3, PAK2, PHB2, RACK1, and RUVBL1	*Training set*: 20 cancers (10 DCIS and 10 early-stage PBC) versus 20 BBL versus 20 AID versus 20 HC	SERPA	0.61; 0.56; 0.55; 0.59; 0.52 (HC versus cancer) 0.67; 0.59; 0.62; 0.61; 0.59 (HC versus early-stage PBC) 0.56; 0.52; 0.50; 0.57; 0.50 (HC versus DCIS)	32; 25; 24; 31; 24% (HC versus cancer) 47; 31; 32; 31; 31% (HC versus early-stage PBC) 24; 27; 18; 29; 18% (HC versus DCIS)	94; 96; 97; 94; 94% (HC versus cancer) 88; 94; 94; 97; 93% (HC versus early-stage PBC) 97; 91; 97; 94; 94% (HC versus DCIS)	0.81 (HC versus cancer) 0.81 (HC versus early-stage PBC) 0.85 (HC versus DCIS)	62–66% (HC versus cancer) 63–71% (HC versus early-stage PBC) 73–82% (HC versus DCIS)	83–87% (HC versus cancer) 81–84% (HC versus early-stage PBC) 74–82% (HC versus DCIS)	[28]
	*Validation set*: 55 DCIS versus 59 early-stage PBC versus 68 HC	ELISA							

p62, p53, c-myc, survivin, p16, cyclin B1, cyclin D1, and CDK2	*Training set*: 1 cancer versus 4 HC	Miniarray	Frequency of AABs to eight cancer-associated antigens: 12.2; 12.2; 22; 22; 12.2; 17.1; 17.1; 9.8% (breast cancer) and 1.2; 2.4; 0; 1.2; 2.4; 1.2; 2.4; 1.2% (HC)			Frequency of AABs to any eight antigens: 61% (breast cancer) and 11% (HC)			[29]
	*Validation set*: 41 cancers versus 82 HC	ELISA	—	22% (c-myc)	100% (c-myc)	—	61%	89%	

hnRNPF and FTH1	*Training set*: 5 cancers versus 5 HC	SEREX	0.725; 0.686	84.2; 81.2%	60.8; 56.1%	0.816	91.1% 89.3% (when combined with CA15-3)	72% 93.8% (when combined with CA15-3)	[30]
	*Validation set*: 155 breast cancers versus 155 HC versus 40 others cancers	ELISA							

RBP-Jk, HMGN1, PSRC1, CIRBP, and ECHDC1	*Training set*: 20 DCIS versus 20 early-stage PBC	Microarray	0.57; 0.58; 0.51; 0.51; 0.54	62.7; 59.3; 16.9; 80.6; 59.3%	57.4; 54.1; 93.4; 31.8; 60.7%	0.794	83.3–86.1%	72.7–75%	[35]
	*Validation set*: 61 DCIS versus 59 early-stage PBC	ELISA							

TAA: tumor-associated antigens; AABs: autoantibodies; AUC: area under curve; DCIS: ductal carcinoma *in situ*; PBC: primary breast cancer; HC: healthy control; AID: autoimmune disease; BBL: benign breast lesions; Ref: references.
